# Supplementary material for: “It just seemed like a perfect storm”: A multi-methods feasibility study on the use of Facebook, Google Ads, and Reddit to collect data on abortion-seeking experiences from people who considered but did not obtain abortion care in the United States
Source: PLoS One. 2022 Mar 3;17(3):e0264748. doi: 10.1371/journal.pone.0264748 (PMC8893629; doi:10.1371/journal.pone.0264748)
Supplement: S1 File — (DOCX) [file pone.0264748.s001.docx]

**Brief survey**

Study ID: _____

1. How did you find out about this study?
   - Facebook
   - Google
   - Reddit
   - Web forum
   - Friend sent me the link
   - Other (Please tell us): ___________________________________
2. How many pregnancies have you had in the past five years (since 2013), including a current pregnancy?
   - 0
   - 1
   - 2
   - 3
   - 4
   - 5+

Display Q3 if Q2=0

1. You answered that you have not been pregnant in the last five years and are not currently pregnant. Is this information correct?
   - Yes
   - No

If answer to Q3==”Yes”, skip to Q14

Display Q4 if Q3=“No”

1. How many pregnancies have you had in the past five years (since 2013), including a current pregnancy?
   - 0
   - 1
   - 2
   - 3
   - 4
   - 5+
2. If (Q2 and Q4!=0) For how many of these pregnancies in the past five years did you consider having an abortion, even for just one second?
   - 0
   - 1
   - 2
   - 3
   - 4
   - 5+
   - All
3. If it had been available to you, for how many pregnancies in the past five years could abortion potentially have been the best option?
   - 0
   - 1
   - 2
   - 3
   - 4
   - 5+
   - All
   - I’m currently pregnant and still deciding whether to have an abortion
4. For how many of these pregnancies in the past five years did you obtain an abortion?
   - 0
   - 1
   - 2
   - 3
   - 4
   - 5+
   - All
   - I’m currently pregnant and still deciding whether to have an abortion
   - Prefer not to answer

Display Q8 if Q6!=”I’m currently pregnant and still deciding whether to have an abortion” OR Q7!=”I’m currently pregnant and still deciding whether to have an abortion”

1. If you considered an abortion for at least one of these pregnancies but did not obtain an abortion for that pregnancy, what were some of the reasons you did not obtain an abortion? Please select all that apply.
   - Could not locate a nearby abortion provider
   - Could not pay for an abortion
   - Could not get time off work for the abortion
   - Could not find childcare for the time needed for the abortion
   - Was too far along in pregnancy when I discovered the pregnancy
   - Personally opposed to abortion
   - Worried that someone would judge me or think less of me if I got an abortion
   - Not listed (please specify): ____________________

Display Q9 if Q6!=”I’m currently pregnant and still deciding whether to have an abortion” OR Q7!=”I’m currently pregnant and still deciding whether to have an abortion”

1. If you considered an abortion for at least one of these pregnancies but did not obtain an abortion for that pregnancy, can you please tell us in your own words about why you did not obtain the abortion?
   - [Free text field]
2. Please tell us in your own words what you mean when you say you “considered” an abortion for one or more pregnancies. (i.e. – Does that mean that you wanted an abortion for that pregnancy? Thought about abortion? Talked to others about abortion? Something else?)
   - [Free text field]

Display Q11 if Q6>=1

1. Please tell us in your own words why you feel that abortion could have been the best option for you for one or more of your pregnancies.
   - [Free text field]

Display Q12 if Q6=”I’m currently pregnant and still deciding whether to have an abortion” or Q7= “I’m currently pregnant and still deciding whether to have an abortion”

1. You said you are currently deciding whether abortion would be the best option for this pregnancy. What factors are you considering in your decision-making process?

_____________________________________________________

_____________________________________________________

Display Q13 if Q6=”I’m currently pregnant and still deciding whether to have an abortion” or Q7= “I’m currently pregnant and still deciding whether to have an abortion”

1. Have you faced any barriers to accessing abortion services at this point in your pregnancy? Select all that apply.

- Have not been able to locate a nearby abortion provider
- Have not been able to gather funds for an abortion
- Have not been able to find childcare for the time needed for the abortion
- I discovered I was pregnant late in the pregnancy
- I’m navigating my personal views of abortion
- I am worried that someone would judge me or think less of me if I got an abortion
- Not listed (please specify): __________________________________

1. What state do you live in?

__________________

1. How many children do you have currently?

__________________

1. What is your total annual household income (in dollars)?

___________________

1. How many individuals in your household are supported by this income?

___ [Enter number]

1. How would you describe your work status right now? (Select all that apply)

- Employed full time(40 hours or more per week)
- Employed part-time (Up to 39 hours per week)
- Self-employed
- Unemployed
- Student
- Retired
- Homemaker
- Unable to work

1. How would you describe your gender?

- Female
- Male
- Prefer to self-describe _______________

1. What is your current health insurance status?
   - Public/government provided health insurance
   - Private health insurance
   - No health insurance
2. What is your current relationship status?
   - Single (never married)
   - In a relationship, **not** **living** with partner
   - In a relationship, **living** with partner
   - Married or in a civil union
   - Separated/Widowed/Divorced
3. What is the highest level of education you have completed?

- No formal education
- Less than high school
- Some high school, no diploma
- High school graduate
- GED
- Some college
- Associate degree
- Bachelor’s degree
- Master’s degree or higher

1. Are you of Spanish, Hispanic, or Latinx descent?

- Yes
- No

1. Please check one or more categories below to indicate what race(s) you consider yourself to be. (Select all that apply)
   - American Indian or Alaska Native
   - Asian
   - Black or African-American
   - Hispanic or Latinx
   - Middle Eastern or North African
   - Native Hawaiian or Other Pacific Islander
   - Bi-racial/multi-racial/mixed
   - White
   - Another (please specify): _________________________
   - Prefer not to answer
2. During the past month, would you say you had enough money to meet your basic living needs such as food, housing, and transportation?
   - - - All the time
       - Most of the time
       - Some of the time
       - Rarely
       - Never
       - Don't know
3. How many people do you know that you think have had an unwanted pregnancy for which they wanted an abortion but did not get one?

__________

1. To how many people would you feel comfortable giving information about this study? (i.e. – referring them to complete the survey)

___________

Display if <4 responses are “Okay, I’ll send the link.”

28b. Please send this link to anyone you think may be interested in taking part in this study. Take a minute to copy the link now.

- Okay, I’ll send the link
- I don’t want to send this link out to anyone in my network

Thank you for completing the survey! Please click here [enter link] if you would like to be entered into the drawing for a $50 Amazon Gift Card.

Please enter the email address you would like the gift card sent to if you win this raffle.

____________________________

Please re-enter the email address you would like the gift card sent to if you win this raffle.

_________________________

PLACEHOLDER FOR TEXT FROM INFORMED CONSENT TO SHARE CONTACT INFO
