# Supplementary material for: “It just seemed like a perfect storm”: A multi-methods feasibility study on the use of Facebook, Google Ads, and Reddit to collect data on abortion-seeking experiences from people who considered but did not obtain abortion care in the United States
Source: PLoS One. 2022 Mar 3;17(3):e0264748. doi: 10.1371/journal.pone.0264748 (PMC8893629; doi:10.1371/journal.pone.0264748)
Supplement: S2 File — (DOCX) [file pone.0264748.s002.docx]

**Semi-Structured In-Depth Interview Guide**

**Introductory Script- see consent form**

**A few simple questions to get us started that will help us understand how your experiences compare to other people from varied backgrounds:**

- Do you mind telling me your age now?
- What is the highest level of education you have completed?
- How do you describe your ethnicity?
- How do you describe your gender?
- What is your current zip code?
- How old were you at the time of your first pregnancy?
- How many children are you currently parenting? (your own (via birth or adoption), or a partner’s)
- Where did you hear about this study?
  - Probe: How did you feel about clicking on the ad?
- Why do you want to participate in this study?

1. **How many times have you been pregnant?**

Probes:

- - How many of these were in the last five years (since 2013)?
  - How many of these pregnancies happened at a time you were trying to get pregnant? (Specify those in the last 5 years, versus all)
  - How many when you were not trying to get pregnant? (Specify those in the last 5 years, versus all)

1. **In the online survey you recently completed, you indicated that for at least one of these pregnancies, if abortion had been available to you, it may have been the best option for resolving that pregnancy. For how many of these pregnancies do you feel that might be true?**
2. **Thinking back to the most recent pregnancy for which abortion might have been the best option, walk me through the experience of discovering that pregnancy until you decided what to do about the pregnancy.**

Probes:

- How old were you?
- Tell me more about how you were feeling when you found out you were pregnant.
- Did anything about your personal circumstances at the time make finding out you were pregnant more or less difficult?
- Were you using birth control at that time?
- At what point in your pregnancy did you find out you were pregnant?
- Did you talk to anyone about the pregnancy? Who did you talk to? And how did they respond?
- How/where did you learn about your options for keeping or terminating the pregnancy?
- How did you navigate deciding what option you would pursue for this pregnancy?

1. **Please tell me in your own words what you mean when you say that you “considered” abortion for this pregnancy.**

Probes:

- Do you mean you thought about abortion as an option, but did not want one?
- Do you mean that you wanted an abortion?
- Something else?
- If you had to use a word other than “considered”, what word would you use to describe your thoughts about abortion in relation to the pregnancy?

1. **We’d like to ask you more about the process you went through considering an abortion for this pregnancy. What were the factors that ultimately led you to *not* have an abortion for the pregnancy?**

Probes:

- - If you had to name specific barriers to abortion care that you experienced, what were they?
  - What did you know about abortion at the time of your pregnancy?
  - What did you know about other pregnancy options (prenatal care, adoption)?
  - How did your personal views on abortion play into this, if at all? Did these change at all throughout the decision making process?
  - How did the availability of abortion services play into your decision, if at all?
    - Did you know where you could go to get an abortion?
    - How near or far was the nearest place? What would this have meant for you in terms of transportation, time off work, child care, etc?
- Did you have an idea how much an abortion would cost? How did the cost of an abortion play into your decision, if at all? Was this influenced by insurance?
  - Was there any information on the procedure that played a factor in your decision?
  - Where there any other circumstances you think that played a role? (Probe for laws, waiting periods, gestational age, etc)
  - Was the opinion of anyone else important to you? If yes, who?
  - Did anyone talk to you about abortion? If so – how? Did you get a sense that they had opinions (moral or emotional or otherwise) about abortion as an option? How did this impact you?
  - At the time of your pregnancy, was there any specific information you wanted to know about your options for the pregnancy?
  - Probe: If participant reports having considered abortion for more than one pregnancy, ask if there was anything different about those prior pregnancies as compared to the most recent one that they would like to share.

1. **So, to summarize, what would you say was the main reason you did not get an abortion for that pregnancy?**
2. **What would have had to be different for you to have been able to get an abortion?** (Only give examples if participant seems confused: “For instance, if the abortion had cost less money, or been closer to your home, or if your partner had been supportive, or any other reason”)
3. **Thank you. Now, thinking back on your experience during this pregnancy, is there anything else you’d like to share with us about how you navigated the different options available to you? Is there anything you would do differently now?**

**Closing Script:** That is all of the questions that I have on my end. Is there anything else that you would like to talk about? Do you have any questions, about what we discussed today, or the study more generally? *If no, or after addressing further questions*: I want to thank you again for taking the time to talk with me today. We are so grateful to you for your time. If you have any other questions that come up, please feel free to email or call us at any time using the contact information we provided for you. You will receive a gift card at the email address you provided.
