# Supplementary material for: “It just seemed like a perfect storm”: A multi-methods feasibility study on the use of Facebook, Google Ads, and Reddit to collect data on abortion-seeking experiences from people who considered but did not obtain abortion care in the United States
Source: PLoS One. 2022 Mar 3;17(3):e0264748. doi: 10.1371/journal.pone.0264748 (PMC8893629; doi:10.1371/journal.pone.0264748)

PLOS ONE

“It just seemed like a perfect storm of everything coming together so that I couldn’t have the [abortion]”: a multi-methods study on people who considered but did not obtain abortion care in the United States

--Manuscript Draft--

**Manuscript Number:**	PONE-D-20-38906
**Article Type:**	Research Article
**Full Title:**	“It just seemed like a perfect storm of everything coming together so that I couldn’t have the [abortion]”: a multi-methods study on people who considered but did not obtain abortion care in the United States
**Short Title:**	A multi-methods study on people who failed to obtain abortion care in the United States
**Corresponding Author:**	Heidi Moseson, PhD, MPH<br>Ibis Reproductive Health<br>OAKLAND, CA UNITED STATES
**Keywords:**	abortion; Facebook; Google Ads; recruitment; Reddit; reproductive health; selection bias; Social media; Stigma; unplanned pregnancy
**Abstract:**	Introduction: Most studies of abortion access have recruited participants from abortion clinics, thereby missing people for whom barriers to care were insurmountable. Consequently, research may underestimate the nature and scope of barriers that exist. We aimed to recruit participants who had considered, but failed to obtain, an abortion to understand their abortion-seeking experiences.<br>Materials and methods: In 2018, we recruited participants from Facebook, Google Ads, and Reddit for an online survey about experiences seeking abortion care in the United States. We conducted in-depth interviews with a subset of survey participants. We completed descriptive analyses of survey data, and thematic analyses of interview data.<br>Results: Overall, 1,191 unique people initiated the study. Among these respondents, 88.9% (n=1,059) reported a pregnancy in the past five years, and 18.2% (n=217) said that abortion would potentially have been the best option for that pregnancy if it had been accessible. After additional screening, 30.4% (n=66) of these respondents reported not having obtained an abortion, nor visited an abortion facility, despite feeling that abortion could have been the best option for a recent pregnancy. A subset of survey respondents (n=14) completed in-depth interviews. Results highlighted multiple, interacting barriers to abortion care, including legal restrictions such as gestational limits and waiting periods that exacerbate financial and other burdens, logistical and informational barriers, as well as barriers to abortion care less frequently reported in the literature, such as a preference for medication abortion.<br>Discussion: These findings contribute to our understanding of the full range of barriers to abortion care that people experience in the United States, and how these barriers intersect to not just delay, but to outright prevent people from obtaining abortion.
**Order of Authors:**	Heidi Moseson, PhD, MPH
	Jane W. Seymour
	Carmela Zuniga
	Alexandra Wollum
	Anna Katz
	Terri-Ann Thompson
	Caitlin Gerdts
**Additional Information:**	
**Question**	**Response**
**Financial Disclosure**	HM, CG received a grant from the Society of Family Planning (grant#: SFPRF11-11) to fund this work (https://societyfp.org/). The funders had no role in study design, data

Powered by Editorial Manager® and ProduXion Manager® from Aries Systems Corporation

Enter a financial disclosure statement that describes the sources of funding for the work included in this submission. Review the submission guidelines for detailed requirements. View published research articles from *PLOS ONE* for specific examples.<br><br>This statement is required for submission and **will appear in the published article** if the submission is accepted. Please make sure it is accurate.<br><br>**Unfunded studies**<br>Enter: *The author(s) received no specific funding for this work.*<br><br>**Funded studies**<br>Enter a statement with the following details:<br>• Initials of the authors who received each award<br>• Grant numbers awarded to each author<br>• The full name of each funder<br>• URL of each funder website<br>• Did the sponsors or funders play any role in the study design, data collection and analysis, decision to publish, or preparation of the manuscript?<br>• **NO** - Include this sentence at the end of your statement: *The funders had no role in study design, data collection and analysis, decision to publish, or preparation of the manuscript.*<br>• **YES** - Specify the role(s) played.<br><br>* typeset	collection and analysis, decision to publish, or preparation of the manuscript.
**Competing Interests**<br><br>Use the instructions below to enter a competing interest statement for this submission. On behalf of all authors, disclose any competing interests that could be perceived to bias this work—acknowledging all financial support and any other relevant financial or non-financial competing interests.<br><br>This statement **will appear in the published article** if the submission is accepted. Please make sure it is accurate. View published research articles from *PLOS ONE* for specific examples.	The authors have declared that no competing interests exist.

**NO authors have competing interests**<br><br>Enter: *The authors have declared that no competing interests exist.*<br><br>**Authors with competing interests**<br><br>Enter competing interest details beginning with this statement:<br><br>*I have read the journal's policy and the authors of this manuscript have the following competing interests: [insert competing interests here]*<br><br>* typeset	
**Ethics Statement**<br><br>Enter an ethics statement for this submission. This statement is required if the study involved:<br><br>• Human participants<br>• Human specimens or tissue<br>• Vertebrate animals or cephalopods<br>• Vertebrate embryos or tissues<br>• Field research<br><br>Write "N/A" if the submission does not require an ethics statement.<br><br>General guidance is provided below. Consult the submission guidelines for detailed instructions. **Make sure that all information entered here is included in the Methods section of the manuscript.**	The Allendale Investigational Review Board reviewed and approved this study (Study ID: Who is Missing 2017-2018). All subjects provided written informed consent.

Powered by Editorial Manager® and ProduXion Manager® from Aries Systems Corporation

**Format for specific study types**<br><br>**Human Subject Research (involving human participants and/or tissue)**<br>• Give the name of the institutional review board or ethics committee that approved the study<br>• Include the approval number and/or a statement indicating approval of this research<br>• Indicate the form of consent obtained (written/oral) or the reason that consent was not obtained (e.g. the data were analyzed anonymously)<br><br>**Animal Research (involving vertebrate animals, embryos or tissues)**<br>• Provide the name of the Institutional Animal Care and Use Committee (IACUC) or other relevant ethics board that reviewed the study protocol, and indicate whether they approved this research or granted a formal waiver of ethical approval<br>• Include an approval number if one was obtained<br>• If the study involved *non-human primates*, add *additional details* about animal welfare and steps taken to ameliorate suffering<br>• If anesthesia, euthanasia, or any kind of animal sacrifice is part of the study, include briefly which substances and/or methods were applied<br><br>**Field Research**<br><br>Include the following details if this study involves the collection of plant, animal, or other materials from a natural setting:<br>• Field permit number<br>• Name of the institution or relevant body that granted permission	
**Data Availability**<br><br>Authors are required to make all data underlying the findings described fully available, without restriction, and from the time of publication. PLOS allows rare exceptions to address legal and ethical concerns. See the PLOS Data Policy and FAQ for detailed information.	No - some restrictions will apply

A Data Availability Statement describing where the data can be found is required at submission. Your answers to this question constitute the Data Availability Statement and **will be published in the article**, if accepted.<br><br>**Important:** Stating 'data available on request from the author' is not sufficient. If your data are only available upon request, select 'No' for the first question and explain your exceptional situation in the text box.<br><br>Do the authors confirm that all data underlying the findings described in their manuscript are fully available without restriction?	
**Describe where the data may be found in full sentences. If you are copying our sample text, replace any instances of XXX with the appropriate details.**<br><br>• If the data are **held or will be held in a public repository**, include URLs, accession numbers or DOIs. If this information will only be available after acceptance, indicate this by ticking the box below. For example: *All XXX files are available from the XXX database (accession number(s) XXX, XXX.).*<br>• If the data are all contained **within the manuscript and/or Supporting Information files**, enter the following: *All relevant data are within the manuscript and its Supporting Information files.*<br>• If neither of these applies but you are able to provide **details of access elsewhere**, with or without limitations, please do so. For example:<br><br>*Data cannot be shared publicly because of [XXX]. Data are available from the XXX Institutional Data Access / Ethics Committee (contact via XXX) for researchers who meet the criteria for access to confidential data.*<br><br>*The data underlying the results presented in the study are available from (include the name of the third party*	Data cannot be shared publicly because of identifiability concerns given nature of questions asked, and confidentiality promises made during the informed consent process. De-identified and pared down data can be made available to researchers who meet the criteria for access to confidential data and provide a reasonable request.

Powered by Editorial Manager® and ProduXion Manager® from Aries Systems Corporation

and contact information or URL).<br>• This text is appropriate if the data are owned by a third party and authors do not have permission to share the data.<br><br>* typeset	
Additional data availability information:	

Manuscript
Click here to access/download;Manuscript;WAWM_MixedMethods_Manuscrip

"It just seemed like a perfect storm of everything coming together so that I couldn't have the [abortion]": a multi-methods study on people who considered but did not obtain abortion care in the United States

Heidi Moseson[1,#a,*], Jane W. Seymour[2], Carmela Zuniga[2], Alexandra Wollum[1], Anna Katz[1], Terri-Ann Thompson[2], Caitlin Gerdts[1]

[1] Ibis Reproductive Health, Oakland, California, United States of America

[2] Ibis Reproductive Health, Cambridge, Massachusetts, United States of America

[#a]Current Address: Ibis Reproductive Health, Oakland, California, United States of America

[*]Corresponding author

Email: hmoseson@ibisreproductivehealth.org (HM)

Abstract

Introduction: Most studies of abortion access have recruited participants from abortion clinics, thereby missing people for whom barriers to care were insurmountable. Consequently, research may underestimate the nature and scope of barriers that exist. We aimed to recruit participants who had considered, but failed to obtain, an abortion to understand their abortion-seeking experiences.

Materials and methods: In 2018, we recruited participants from Facebook, Google Ads, and Reddit for an online survey about experiences seeking abortion care in the United States. We conducted in-depth interviews with a subset of survey participants. We completed descriptive analyses of survey data, and thematic analyses of interview data.

Results: Overall, 1,191 unique people initiated the study. Among these respondents, 88.9% (n=1,059) reported a pregnancy in the past five years, and 18.2% (n=217) said that abortion would potentially have been the best option for that pregnancy if it had been accessible. After additional screening, 30.4% (n=66) of these respondents reported not having obtained an abortion, nor visited an abortion facility, despite feeling that abortion could have been the best option for a recent pregnancy. A subset of survey respondents (n=14) completed in-depth interviews. Results highlighted multiple, interacting barriers to abortion care, including legal restrictions such as gestational limits and waiting periods that exacerbate financial and other burdens, logistical and informational barriers, as well as barriers to abortion care less frequently reported in the literature, such as a preference for medication abortion.

Discussion: These findings contribute to our understanding of the full range of barriers to abortion care that people experience in the United States, and how these barriers intersect to not just delay, but to outright prevent people from obtaining abortion.

Keywords: abortion; Facebook; Google Ads; recruitment; Reddit; reproductive health; selection bias; social media; stigma; unplanned pregnancy

Introduction

In the United States, people have difficulty accessing abortion care because of a range of barriers, including cost of services and ancillary expenses; gestational age restrictions; legal restrictions; long distances to care; and lack of abortion-related information.[1-14] Although these barriers have negative consequences individually,[9] people seeking abortion often experience them in combination, which compounds their effect on access to care.[15] These intersecting barriers, which result in higher costs, may be particularly burdensome, as the majority of US abortion patients are low-income or live below the Federal Poverty Level.[14]

Although many studies have examined barriers to abortion, most recruited people from abortion-providing facilities, excluding people for whom barriers to care were insurmountable. As a result, existing research may underestimate the extent to which certain factors act as barriers to abortion, and there may be unidentified barriers to care or interactions between barriers. Recognizing the limitation of recruiting only from abortion clinics, a study in Louisiana and Maryland concluded that recruitment in prenatal clinics is a feasible way to find people who considered, but did not obtain, an abortion;[16] approximately one-third of participants considered, but did not obtain an abortion, citing personal preferences and policy-related barriers. Another recruited currently pregnant participants searching for information on abortion from Google Ads

The harms of not obtaining wanted abortion care are well established,[17-22] yet, without understanding the full range and impact of barriers to abortion, public health practitioners and policymakers cannot develop appropriate interventions to improve access. With this multi-methods study, we aimed to recruit a narrow population of people who have been left out of prior research on abortion access: namely, people who had considered, but not obtained an abortion, nor made it to an abortion clinic, and to assess their experiences with accessing abortion care. We hypothesized that online (as opposed to clinic-based) recruitment methods would facilitate identification of people that had experienced multiple barriers to abortion care, of greater magnitude than those previously described in the literature.

Materials and methods

Recruitment

Between August 15 and September 15, 2018, we recruited for a brief online survey through advertisements on Facebook, Google Ads, and two Reddit threads (birth control and menstruation). The one-month recruitment period mirrored a recent abortion-related study that recruited using GoogleAds.[23] A digital marketing firm, BUMP Recruitment, managed the posting and purchasing of Facebook and Google Ads advertisements. Study authors managed the Reddit campaigns. English and Spanish advertisements read: "Complete a 5 minute survey about unplanned pregnancy and be entered to win $50 gift card" and offered a link to complete an "Unplanned Pregnancy Study." The advertisements led to a study website with additional study information and a link to the eligibility screener. If eligible, individuals were offered the opportunity complete the short online survey. At survey completion, participants could indicate their interest in an in-depth interview. More details on study recruitment have been described elsewhere.

Survey

To recruit the narrowly defined population of interest, the survey screener identified eligible participants: aged 15 to 49 years old, English or Spanish speaking US residents, who self-identified as neither having visited an abortion clinic nor obtained an abortion for a pregnancy in the past five years. Eligibility questions included: "Did you consider abortion for any of these pregnancies, even for just one second?" and "If it had been available to you, could abortion have potentially been the best option for any of these pregnancies?" An individual needed to respond "yes" to both of these questions to be eligible. The survey was administered via Qualtrics (Qualtrics, Provo, UT) and included up to 28 open- and closed-ended questions about experiences with unwanted pregnancy, considering abortion, obtaining abortion care, and sociodemographic characteristics. Adaptive questioning reduced the number of questions based on responses; questions were displayed between three and 12 screens. Respondents could not go back to revise responses. Study team members pre-tested the questionnaire's technical functionality before study launch.

Respondents reported number of pregnancies in the last five years, for how many pregnancies they considered abortion, for how many pregnancies abortion could have been the best option, and barriers they faced when seeking abortion care. In addition to selecting from pre-specified barriers, participants could list barriers and were asked, "Please tell us in your own words about why you did not obtain the abortion." Participants who completed the survey were entered into a $50 gift card raffle. Because this study was partially designed to assess the efficiency of the three tested recruitment methods, we did not set a target sample size, and instead aimed to measure if each method could reach the narrowly defined target population.

We conducted descriptive analyses of data from closed-ended survey questions using Stata version 15.1 (Stata, StataCorp, College Station, TX), and summarized open-ended question responses. Where appropriate, closed-ended responses were recoded based on open-ended responses. We report results in accordance with the Checklist for Reporting Results of Internet E-Surveys (CHERRIES).[24]

In-depth interviews

Survey participants interested in interview participation provided their name or pseudonym, email address or phone number, and preferred language. Five cisgender women trained in in-depth interviewing, conducted all 30-60 minute interviews. Participants who completed the interview received a $25 gift card. As a formative study, we aimed to capture a handful of experiences to identify areas for further research, rather than a pre-specified sample size. We conducted an in-depth interview with all interested participants who responded to investigator outreach.

Using a semi-structured guide, interviewers asked participants about: their experiences with pregnancy; circumstances, emotions, and decision-making processes related to their most recent pregnancy for which abortion may have been the best option; factors or barriers that led them to not have an abortion; and, if abortion was considered for more than one pregnancy, any differences in circumstances compared to their most recent pregnancy for which they considered abortion; and sociodemographic characteristics. Interviews were conducted and audio-recorded via a secure online

platform, and subsequently transcribed. We analyzed de-identified transcripts thematically, using an iteratively adapted codebook, which initially contained a priori identified codes. To ensure consistency, two transcripts were coded independently by three reviewers, and discrepancies were resolved prior to coding the remaining transcripts. We coded interviews in Dedoose (Dedoose Version 8.1.8 (2018) Los Angeles, CA).

The survey and in-depth interview guide were designed to complement one another. We analyzed survey data prior to interview data. We note convergence between survey and interview findings in the results. Along with quotes from open-ended survey questions or interviews, we include participants' age, self-reported race or ethnicity, and U.S. Census Region for context.

Ethical review

This study was approved by the Allendale Investigational Review Board. All participants gave written consent to participate in the survey, and verbal consent to participate in the interview.

Results

Survey results

<u>Participant characteristics</u>

Overall, respondents submitted 1,254 eligibility screener completions, 1,191 of which came from unique internet protocol addresses. Among these respondents, 88.9% (n=1,059) reported a pregnancy in the past five years, and 18.2% (n=217) said that abortion would potentially have been the best option for that pregnancy if it had been accessible. After additional screening for age, residence in the US, and visits to abortion providing facilities, 98 eligible participants began the full survey, of whom 90 (92%) completed it (Figure 1). We excluded 11 surveys (11%) from respondents who reported that the main reason they had not obtained an abortion was because they had a miscarriage, and an additional 13 surveys (14%) from respondents who were currently pregnant, as it was possible that some would go on to obtain an abortion. The final population of interest included 66 participants from 30 states (Figure 2).

Figure 1. Study flow diagram displaying participant pathways from screening to survey to interview

Figure 2. Representation of the 30 states included in the study sample

Blue = one or more respondents from this state participated in the study;
Grey = no respondents from this state participated in the study

Participants were 29 years old on average, the majority identified as cisgender women and white, and eight (12%) as Hispanic or Latinx. Nearly two-thirds lived in the Midwest or South Census regions, and 53 (80%) had health insurance (Table 1).

Table 1. Characteristics of eligible survey participants (n=66)

Participant Characteristics	*n*	*%*
Gender identity		
Cisgender Woman	57	86
Missing	9	14
Age		
Average, SD	29	6
Minimum, maximum	19	44
Missing	0	0
Census region*		
Northeast	5	8
South	27	41
Midwest	16	24
West	9	14
Missing	9	14
Relationship status		
Single	9	14
In a relationship	21	32
Married/civil union	26	40
Separated/widowed/divorced	3	5
Missing	9	14
Education		
Less than high school graduate	3	5
High school graduate or GED	11	17
Some college or Associates degree	27	41
Bachelor's degree or more	10	15
Missing	9	14
Race*		
American Indian or Alaska Native	4	6
Middle Eastern or North African	1	2
Native Hawaiian or Pacific Islander	1	2
White	49	74

Missing	13	20
Hispanic		
Yes	8	12
Missing	9	14
Proportion of the time you had enough money to meet basic living needs last month		
All/most of the time	35	53
Some of the time/rarely/never	22	33
Missing	9	14
Current insurance status		
Public/government	31	47
Private	22	33
Uninsured	4	6
Missing	9	14

* https://www2.census.gov/geo/pdfs/maps-data/maps/reference/us_regdiv.pdf
** Participants could select more than one response

Barriers to abortion care

From the multiple choice list of barriers to abortion, all participants reported at least one listed barrier, and over half (n=34, 52%) experienced two or more, with 19 (29%) reporting three or more. The most commonly reported barriers were ability to pay (56%), concern about judgement (45%), and ability to locate a provider (35%) (Table 2).

Table 2. Barriers to abortion access (N=66)

	Past pregnancies
	n (%)
Could not pay	37 (56)
Worried about judgment	30 (45)
Could not locate nearby provider	22 (33)
Could not get time off from work	9 (14)
Too far in pregnancy	6 (9)
Personally opposed to abortion	6 (9)
Could not find childcare	3 (5)
Another barrier	14 (21)

Note: columns may not total 100%, as participants could select more than one response.

In open-ended responses, 13 (19.6%) participants described additional reasons they did not obtain an abortion. Twelve noted personal reasons, including eight who chose to continue the pregnancy, two who would “hate” themselves for having an abortion, one who “wouldn’t be able to handle it,” and one

who felt it was wrong to terminate because they were marrying the other person involved in the pregnancy. One respondent from the South (white, 28 years old) encountered an informational barrier to abortion care stating that they read “information online that I now know to be completely false but affected my emotional decisions at the time.”

In-depth interview results

Participant characteristics

We conducted 14 interviews. Ten interviews were included; we excluded four interviews from participants who reported a miscarriage as the primary reason that they did not obtain abortion. Nine participants disclosed their age at the time of their most recent pregnancy for which they considered abortion; ages ranged from 19 to 43 years. All participants discovered their pregnancy at or before 12 weeks’ gestation (Table 3).

Table 3. Characteristics of eligible in-depth interview participants (n=10)

	N
Gender identity	
Cisgender woman	10
Age at time of interview, years	
Average	30
Minimum, maximum	20, 45
Census region*	
Northeast	0
South	4
Midwest	4
West	2
Education	
Less than high school graduate	
High school graduate or GED	2
Some college or Associates degree	3
Bachelor’s degree or more	5
Number of children	
0	0
1	4
2	2
3	3
6	1
Race*	
American Indian or Alaska Native	1
Native Hawaiian or Other Pacific Islander	1

White	8
Hispanic	
Yes	3
Age at first pregnancy	
17-19	4
20-22	4
23-25	2
Number of pregnancies in the past 5 years	
1	2
2	3
3	3
4	2
Number of pregnancies in the past 5 years for which abortion could have been the best option	
1	8
2	2

** https://www2.census.gov/geo/pdfs/maps-data/maps/reference/us_regdiv.pdf*
*** Participants could select more than one response*

Barriers to abortion care

Consistent with survey results, participants described multiple barriers that prevented them from obtaining abortion care. In most cases, two or more barriers amplified one another.

Financial barriers. All but one participant mentioned the cost of abortion as a barrier to care. Participants described low hourly wages and a lack of savings that precluded their ability to pay for care, partners who did not or would not contribute to abortion costs, and having to decide between paying essential monthly bills and paying for an abortion. Some noted the irony of cost being a barrier, given the low cost of abortion relative to parenting. One participant from the Midwest (white, 17 years old at time of pregnancy) highlighted how lack of insurance coverage for abortion, possibly as a result of policy restrictions on public funding for abortion, made the cost of abortion too high:

> *[…] insurance did not cover [abortion] at all, so everything would be out-of-pocket. And any complications that arose from it would also not be covered by insurance. It sounds dumb at the time, but it was more expensive for me to go through with an abortion than it was to just keep the baby, which after you have the baby, obviously it's a lot more expensive than just terminating a pregnancy. But it's just one of those things that if you don't have the [money] upfront for it, then they can't really do it.*

Participants also described how financial costs layered on top of other barriers, including a lack of information about abortion cost, gestational limits, stigma from partners or family, and a need for time off from work and/or childcare. Most participants did not have accurate information on the cost of abortion, either because clinics would not quote a price over the phone or they found conflicting information in online forums and Internet search results. While some participants were aware of abortion funds, one did not reach out to a fund due to uncertainty as to how it might work with her insurance. This lack of information further complicated participants' planning. Several described that financial challenges were exacerbated by the gestational limit on abortion in their state, meaning that they had a short window of time to gather the needed funds, and restrictions on public funding for abortion.

Policy-related barriers. Nearly all participants discussed policy-related barriers to care that affected their abortion-seeking experience; in particular, waiting periods, gestational limits, and restrictions on public funding for abortion. One participant explicitly stated that abortion-related laws acted as a "roadblock" to obtaining abortion, because she could not get information she needed to navigate the conflicting and varied restrictions by state. As a result of gestational limits, some participants felt time pressure to decide whether abortion was right for their pregnancy, knowing that they only had a few weeks from discovery of the pregnancy until the gestational limit for abortion in their state. In some cases, participants' understanding of the gestational limit in their state was not accurate, but still influenced their decision-making. One participant from the Midwest (Pacific Islander, 25 years old at the time of pregnancy) incorrectly believed she was only days away from the gestational limit, so ended up continuing the pregnancy because she could not make the decision quickly enough:

> *So I just wish that I had a little bit more time to think about it [...] – when I was on week 11 of the pregnancy we were really on the fence about it and I just wish I had more time to think about it.*

Another participant said that she and her partner could not obtain enough money for the abortion before the gestational limit. If that policy had not been in place, she felt she could have raised enough money for the abortion; because she experienced both barriers –cost and the gestational limit – abortion became unobtainable. Another participant described the mandatory counseling and waiting period laws in her

state as a deterrent to seeking abortion care. To comply with the required additional clinic visit, this participant would have had to pay for an overnight stay in a hotel, take more time off from work, and cover childcare costs.

Logistical barriers. Many participants mentioned logistical barriers to abortion, such as an inability to take time off work or requirements to disclose to their employer the reason for time off, an inability to find childcare, or time required for travel. Participants discussed how policy and financial barriers exacerbated the barriers imposed by these logistical factors. For instance, the need for time off work and childcare was often compounded by large distances to the nearest abortion clinic; many reported drive times of 3.5-4 hours to the nearest facility and needing at least two nights in a hotel, which increased the ancillary costs of abortion and made it financially unobtainable. A participant from the South (white, 25 years old at the time of her pregnancy) described multiple such barriers: the logistical barrier of a four hour drive across state lines to the nearest abortion provider; the social barrier of anti-abortion stigma from her partner, sister, and other family members; the logistical barrier of her partner traveling for work for months at a time, leaving her alone with her children; and an additional logistical barrier of the inability to obtain childcare for her children, one of whom had special needs:

> *I still look at my [child] all the time and think like I wish I had – I know this sounds really awful – I just wish I would have tried harder [to have the abortion]. I wish I would have offered someone some money to watch my [other child] or tried to convince someone – tried to plead with my sister or someone to come out to watch him. …at the time, I did the best I could do, but … it just seemed like a perfect storm of everything coming together so that I couldn't have the procedure done.*

Informational barriers. Participants described a lack of information about abortion. The most frequently reported information gaps included not knowing the cost of abortion care, details about the procedure and what to expect, and where or how to find an abortion-providing facility. Other information gaps included knowledge of gestational age eligibility, any long-term health effects of abortion, and whether legal restrictions existed in their state. Many interviewees did not have all of the information

about abortion they wanted. The information that participants obtained came from online searches and forums, and the experiences of friends. Even health care providers were not always a trusted or reliable source of information; several participants mentioned frustration with providers who never acknowledged abortion as an option, omitted information about abortion from counseling, or explicitly refused to provide information based on their personal opposition to abortion. One participant from the Midwest (white, 17 years old at the time of her pregnancy) described such an interaction, saying:

> *I mean, my doctor gives me every sort of treatment option for like my depression. She gave me options for hypothyroidism. She's given me options for everything. But whenever it came to pregnancy it was 'Congratulations,' and that was it…. They just kind of skated on by and just figured that I was keeping the baby, and that was that.*

Relatedly, two participants received counseling from Crisis Pregnancy Centers (CPCs) without knowing that CPCs did not provide abortions, and reported confusion as to why abortion was not mentioned. In these cases, social stigma toward abortion and assumptions about pregnancy desires directly influenced participants' ability to access thorough and accurate information about abortion. Notably, two respondents stated that they might have made a different choice about their pregnancy had they known more about the cost of abortion, where they could obtain care, and up to what gestational age abortion was available.

Stigma barriers. Participants discussed strong perceived and felt abortion stigma from their partners, families, communities, and health care providers. The desire to avoid judgment and social ostracism delayed participants' decision-making past gestational limits, or deterred them from seeking abortion. Participants described internalized stigma that stemmed from a religious upbringing and was perpetuated by their communities. This interplay of individual and community stigma complicated and lengthened participants' decision-making, and restricted who they could approach for financial and informational support. A participant from the South (Hispanic, 25 at the time of the pregnancy) referenced financial barriers, lack of knowledge, and internalized stigma as barriers that prevented her from obtaining an abortion:

And just how do I drum up money for an abortion? Do I pay my rent or do [I] pay for an abortion? Because my understanding of it was that it was [a] rather expensive option. So I guess I feel like I may have been misinformed about the price at the time, but I pretty much just decided based on I guess social stigmas I had placed on myself; that I should have a baby who is now a toddler who is screaming outside of the door.

Abortion method preference. Some respondents stated that medication abortion was the only acceptable method of abortion for them. In particular, three participants described an aversion to surgical abortion. This preference made participants hesitant to seek abortion care; for some, policy- and information-related barriers intersected with abortion method preferences because they were afraid of being beyond the gestational limit for medication abortion, while for others information-related barriers intersected with preferences because of a misconception that a surgical procedure would be painful for the fetus. One participant from the South (Hispanic, 19 years old at the time of her pregnancy) highlighted how policy, information gaps, and method preference barriers interacted, saying:

…because I didn't want to get an abortion past 12 weeks - or I think is it eight weeks that you can do the pill? I didn't want to go past the pill mark basically, because I don't think I could have gone through further in a pregnancy with the secondary option of abortion…So I just knew that for me, I had to make a choice within the time period for the pill - for the abortion pill.

<u>Consequences of failure to obtain abortion</u>

Across interviews, participants described varied ways in which carrying an unplanned pregnancy to term and raising an unexpected child impacted their lives. Some described shifts to education plans and work ability, and others described emotional and social consequences, including for their relationship with the child. One participant from the South (white, 25 years old at the time of pregnancy) described difficulty in bonding with the pregnancy and the baby:

"Sometimes it's really hard to talk about because my – the one that I wanted the abortion for, he's 2 and he's running around the house right now. […] I never bonded with him during my pregnancy because I just didn't want him. And it took a lot of – it was really a strain to try to

bond with him after he was born. Even when I went into labor with him, I almost had him in the parking lot because I just didn't even want to go in the hospital and do it all. I didn't want to feed him and I didn't want to hold him.[…] I just didn't want to do it all over again."

Discussion

In this multi-methods study, an understudied population – people who failed to obtain abortion or even visit an abortion facility, despite feeling it was the best option for a recent pregnancy - described multiple, intersecting barriers to abortion care which ultimately deterred them from seeking, or prevented them from obtaining, a wanted abortion in the United States. Some reported barriers were consistent with those reported in studies that recruited patients from abortion clinics, including inability to pay for the abortion; long distances to the nearest provider; logistical difficulties; legal restrictions on abortion; and abortion stigma.[1-10, 12, 15, 25] Another barrier identified by this study – medication abortion preference – has not been widely emphasized in the existing literature, while barriers related to inaccurate information about gestational and other legal limits build on findings that explore information gaps related to finding and accessing abortion care.[6,12] Study findings highlight the burden of secondary abortion costs, the shortened timeline for abortion fundraising imposed by gestational limits, and the fear of stigma from health care providers, partners, employers or family.

The proportions of study participants who were insured, were well educated, and had previous pregnancies were high, and nearly half had enough income to meet basic living expenses. Even with these advantages, participants experienced multiple barriers that prevented them from obtaining an abortion. This is cause for concern given the established harms of being denied abortion care,[17-22] including ramifications for socioeconomic status, achieving aspirational one-year plans, perceived stress, future pregnancies, and risk of violence from the man involved in the pregnancy.

Additionally, study findings make clear the reality that barriers to abortion care in the United States intersect to not just delay, but to prevent people from accessing abortion. Targeted interventions could reduce these barriers. To address information gaps, general health care providers – not just those family planning providers – could be trained to discuss pregnancy options, including abortion, for patients

at routine annual exams and in contraception counseling, to ensure that people have abortion information before they need it.[12] Providers' counseling could include information about abortion cost, gestational limits and other restrictions, and abortion funds to help cover the financial and practical costs of care. Similarly, advocates could focus on creating educational campaigns to spread awareness of abortion options and laws, as well as abortion funds, to reduce the number of people deterred from abortion-seeking because of a lack of information or funds. Policy-related interventions could include the expansion of gestational limits for medication abortion, as the scientific evidence confirms that medication abortion is safe at a wider range of gestational ages than for which it is currently approved.[26] This would help ensure that a medication abortion preference is not a barrier to accessing facility-based abortion. Policymakers could also remove waiting period requirements and other laws that result in the need for multiple clinic visits, as these barriers deterred people from seeking care due to the additional monetary and time costs, and fear of stigma from repeated clinic visits. Other policy-related implications include the lack of insurance coverage for abortion, and the resulting financial strain for many participants that exacerbated other barriers.

Limitations

This research has limitations. First, the study population may not be representative of all individuals who considered but did not obtain abortion care. The narrowly defined eligible sample was small, and included no Asian or Black, participants. This lack of racial and ethnic diversity may be a result of the specific recruitment advertisements displayed by social media algorithms, determined by initial engagement. Given established disparities in abortion access, particularly among Black women, this is a major limitation.[27] Furthermore, given that Internet users are not representative of the general population (e.g., [28]), additional work is necessary to recruit for a study that could be generalized to the larger population of US abortion seekers.

Second, recruitment may have been affected by retrospective reporting of pregnancy intention or desire for abortion. We know that retrospective assessment of pregnancy intention changes for those who

are denied a wanted abortion in the clinic setting.[29] However, to be eligible for the current study, individuals had to express interest in a study about unplanned pregnancy and then report that they had considered abortion for “at least one second,” that abortion may have been the best option for more than one pregnancy, and that they did not visit an abortion clinic. Thus, we may have missed people with the experiences of interest, but who do not describe or recall their experiences in alignment with screening questions. Future studies should examine alternative screening questions to assess how such questions affect the sample, or prospective studies should be implemented. Third, there was significant missing data due to participants skipping questions.

We designed this study to identify whether we could recruit a specific population that has rarely been included in prior studies; as a result, we focused narrowly on people who did not obtain an abortion – either clinic-based, or self-managed – and recruited a small sample. Despite the above limitations, this feasibility study successfully utilized three online recruitment strategies to recruit a narrowly defined and understudied population, and the findings presented here can inform future research among larger samples.

Conclusions

This study provides new insight into the experiences of people who considered abortion but failed to reach an abortion facility, much less obtain an abortion. This population has not been widely studied, and the findings presented here underscore the impact that abortion restrictions such as insurance coverage, gestational limits, medication abortion eligibility windows, and two-visit requirements can have in preventing access to essential health care when experienced in concert with financial, information, logistical, and social barriers. Future abortion access studies should sample not just from those who present at abortion-providing facilities, but from all those who consider abortion to better understand the magnitude and scope of barriers to care, and to identify the full range of possible intervention points for dismantling barriers and increasing access to abortion – a safe and essential public health service.[30]

Acknowledgments

We would like to thank our colleagues, Sofia Filippa and Samantha Ruggiero, who together conducted five in-depth interviews for this study, as well as Margot Cohen for support in preparing the manuscript.

References

1. Jones RK, Jerman J. Time to appointment and delays in accessing care among US abortion patients. The Guttmacher Institute, 2016.

2. Gerdts C, Fuentes L, Grossman D, et al. Impact of clinic closures on women obtaining abortion services after implementation of a restrictive law in Texas. American Journal of Public Health. 2016.

3. Karasek D, Roberts SC, Weitz TA. Abortion patients' experience and perceptions of waiting periods: Survey evidence before Arizona's two-visit 24-hour mandatory waiting period law. Women's Health Issues. 2016;26(1):60-66.

4. Fuentes L, Lebenkoff S, White K, et al. Women's experiences seeking abortion care shortly after the closure of clinics due to a restrictive law in Texas. Contraception. 2016;93(4):292-297.

5. Jones RK, Upadhyay UD, Weitz TA. At what cost? Payment for abortion care by US women. Women's Health Issues. 2013;23(3):e173-e178.

6. White K, Grossman D, Turan JM. Experiences accessing abortion care in Alabama among women traveling for services. Women's Health Issues. 2016;26(3):298-304.

7. Upadhyay UD, Weitz TA, Jones RK, Barar RE, Foster DG. Denial of abortion because of provider gestational age limits in the United States. American Journal of Public Health. 2014;104(9):1687-1694.

8. Roberts S, Fuentes L, Kriz R, Williams V, Upadhyay U. Implications for women of Louisiana’s law requiring abortion providers to have hospital admitting privileges. Contraception. 2015;91(5):368-372.

9. Roberts SC, Gould H, Kimport K, Weitz TA, Foster DG. Out-of-pocket costs and insurance coverage for abortion in the United States. Women's Health Issues. 2014;24(2):e211-e218.

10. Foster DG, Kimport K. Who seeks abortions at or after 20 weeks? Perspectives on sexual and reproductive health. 2013;45(4):210-218.

11. Ralph LJ, King E, Belusa E, Foster DG, Brindis CD, Biggs MA. The Impact of a Parental Notification Requirement on Illinois Minors' Access to and Decision-Making Around Abortion. J. Adolesc. Health. Mar 2018;62(3):281-287.

12. Kavanaugh ML, Jerman J, Frohwirth L. "It's not something you talk about really": information barriers encountered by women who travel long distances for abortion care. Contraception. 2019;100(1):79-84.

13. Bearak JM, Burke KL, Jones RK. Disparities and change over time in distance women would need to travel to have an abortion in the USA: a spatial analysis. The Lancet Public Health. 2017;2(11):e493-e500.

14. Jones RK, Ingerick M, Jerman J. Differences in Abortion Service Delivery in Hostile, Middle-ground, and Supportive States in 2014. Women's Health Issues. 2018;28(3):212-218.

15. Jerman J, Frohwirth L, Kavanaugh ML, Blades N. Barriers to Abortion Care and Their Consequences For Patients Traveling for Services: Qualitative Findings from Two States. Perspect Sex Reprod Health. Jun 2017;49(2):95-102.

16. Roberts SCM, Kimport K, Kriz R, Holl J, Mark K, Williams V. Consideration of and Reasons for Not Obtaining Abortion Among Women Entering Prenatal Care in Southern Louisiana and Baltimore, Maryland. Sexuality Research and Social Policy. 2018;16(4):476-487.

17. Harris LF, Roberts SC, Biggs MA, Rocca CH, Foster DG. Perceived stress and emotional social support among women who are denied or receive abortions in the United States: a prospective cohort study. BMC Womens Health. 2014;14:76.

18. Roberts SC, Biggs MA, Chibber KS, Gould H, Rocca CH, Foster DG. Risk of violence from the man involved in the pregnancy after receiving or being denied an abortion. BMC Medicine. 2014;12(1):144.

19. Upadhyay UD, Biggs MA, Foster DG. The effect of abortion on having and achieving aspirational one-year plans. BMC Women's Health. 2015;15(1):102.

20. Biggs M, Upadhyay UD, McCulloch CE, Foster DG. Women's mental health and well-being 5 years after receiving or being denied an abortion: A prospective, longitudinal cohort study. JAMA Psychiatry. 2017;74(2):169-78.

21. Foster DG, Biggs MA, Ralph L, Gerdts C, Roberts S, Glymour MM. Socioeconomic Outcomes of Women Who Receive and Women Who Are Denied Wanted Abortions in the United States. Am J Public Health. 2018;108(3):407-13.

22. Foster DG, Steinberg JR, Roberts SC, Neuhaus J, Biggs MA. A comparison of depression and anxiety symptom trajectories between women who had an abortion and women denied one. Psychol Med. 2015;45(10):2073-82.

23. Jerman J, Onda T, Jones RK. What are people looking for when they Google "self-abortion"? Contraception. 2018;97(6):510-4.

24. Eysenbach G. Improving the quality of Web surveys: the Checklist for Reporting Results of Internet E-Surveys (CHERRIES). J. Med. Internet Res. Sep 29 2004;6(3):e34.

25. Jones RK, Jerman J. How far did US women travel for abortion services in 2008? Journal of Women's Health. 2013;22(8):706-713.

26. Borgatta L, Kapp N, Society of Family P. Clinical guidelines. Labor induction abortion in the second trimester. Contraception. Jul 2011;84(1):4-18.

27. Dehlendorf C, Harris LH, Weitz TA. Disparities in abortion rates: A public health approach. Am J Public Health. 2013;103(10):1772-1779.

28. Groves RM. Three Eras of Survey Research. Public Opin. Q. 2011;75(5):861-871.

29. Rocca CH, Wilson MR, Jeon M, Foster DG. Stability of Retrospective Pregnancy Intention Reporting Among Women with Unwanted Pregnancies in the United States. Maternal and child health journal. 2019;23(11):1547-1555.

30. National Academies of Sciences, Engineering, and Medicine. 2018. The Safety and Quality of Abortion Care in the United States. The National Academies Press. ISBN: 978-0-309-46818-3. DOI: https://doi.org/10.17226/24950.

Figure 1

Click here to access/download;Figure;Figure 1 - Study flow figure.tif

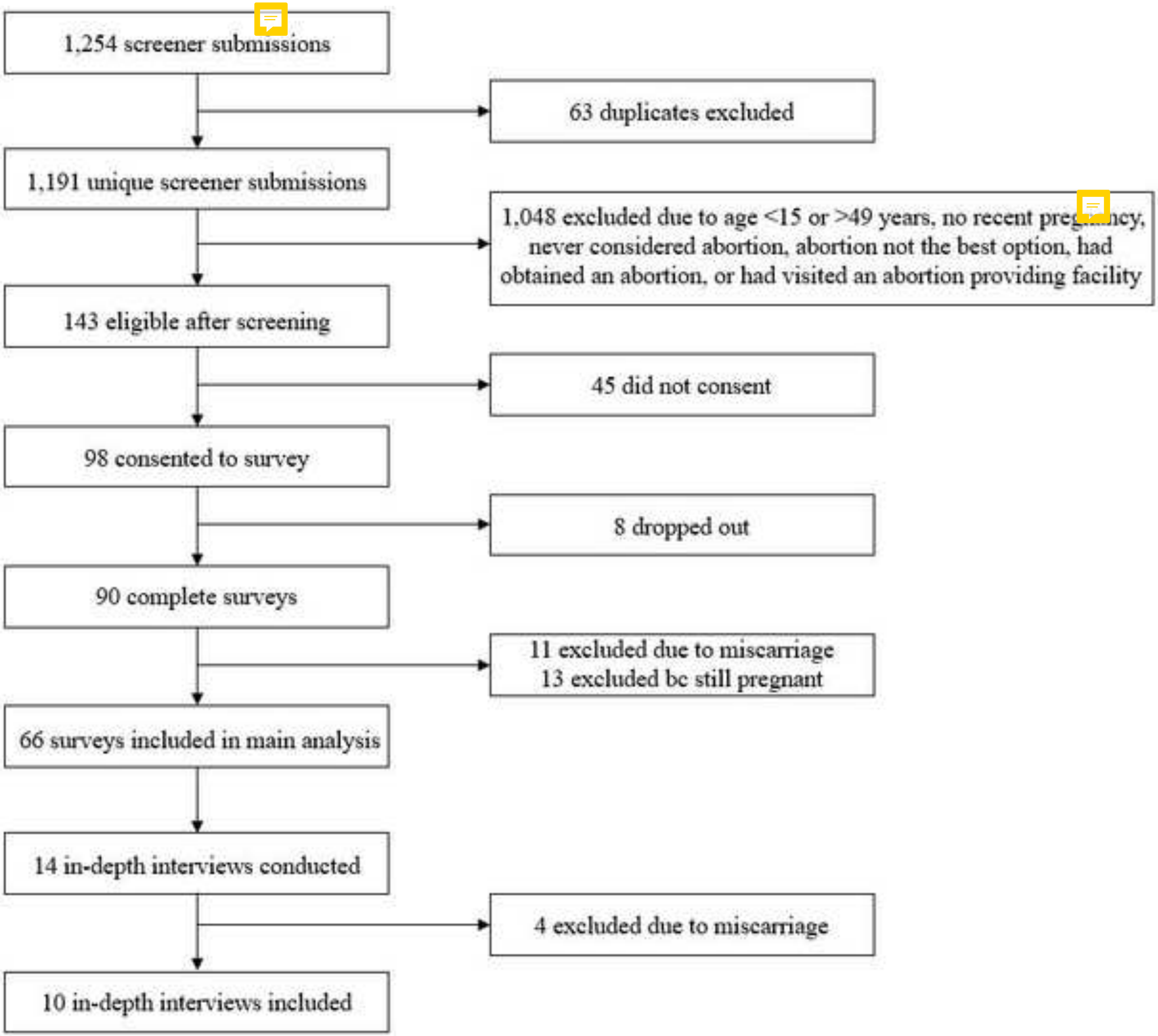

Figure 2

Click here to access/download;Figure;Figure 2 - US Map of included states.tif

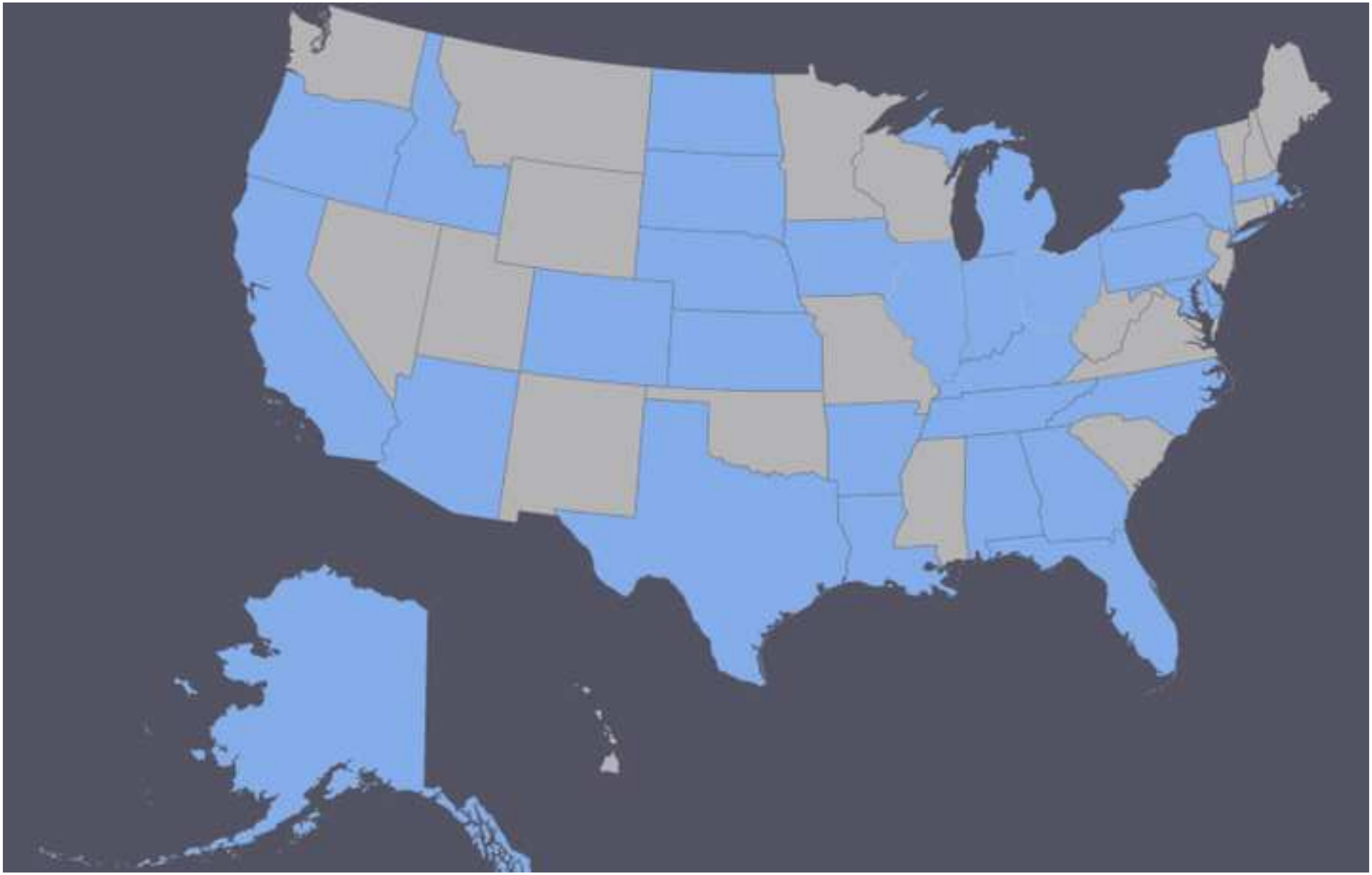

Supplement: Attachment — Submitted filename: PONE-D-20-38906_reviewer.pdf [file pone.0264748.s003.pdf]
